# Constipation

**Published:** 1900-12-22

**Authors:** 


### CONSTIPATION.

Some observations have recently been made in regard to the conditions which conduce to constipation which are certainly worthy of consideration, and all the more so since they seem to explain to a certain extent one of the great difficulties which always stand in the way of securing a proper action by the use of aperient drugs—namely, the tendency to constipation which so constantly follows their cathartic action. According to E. Roos, writing in the "Munchener Medicinische Wochenschrift," an important factor in the production of the normal peristaltic action of the bowels is the stimulation of their coats by the products of the normal flora which inhabit the intestinal tract. Accordingly he cultivated the coli bacillus, and enclosed the culture so obtained in gelatine capsules, which he then coated with collodion and keratin so as to ensure that they should pass through the stomach into the intestines undissolved. These capsules he then administered to several patients during five days, with the effect of relieving them for a couple of weeks of the constipation from which they had previously suffered. The same experiment performed with dead bacilli proved negative. He then used cultures of the bacillus by which lactic acid is produced. These produced increased peristaltic action and caused flatulence, but did not relieve the constipation. Yeast given in the same way, however (in capsules coated as before with keratin), produced no gastric disturbance or flatulence, but after a few days acted as an aperient.

Perhaps it has been too much taken for granted that the action of aperient medicine must as a natural consequence be followed by a reaction in the form of constipation. That this is a common sequence no one can doubt. Nothing, for example, can be more striking than the effect of a calomel and colocynth pill upon a patient unaccustomed to aperients, unless it be the disappointment which follows upon an early repetition of the dose. Yet, unless we are to believe that the aperient has removed some ingredient from the intestinal canal, something which usually acts as an intestinal stimulant, it is by no means easy to explain why such an effect should be produced. It is common enough to hear the matter explained on the hypothesis that it is not the mercurial, but the onflow of the bile thereby set up that causes the aperient effect. Perhaps. But bile is formed too quickly, and the charge in the intestine is too rapidly replaced, to make this explanation entirely satisfactory. If, however, extending the suggestion put forward above, we are to imagine that each portion of the intestine is inhabited by its own special flora, by whose action the successive changes undergone by chyme in its passage along the canal are assisted, and by the products of whose action the intestinal peristalsis is stimulated and maintained, then it is easy enough to see that the taking of an aperient may so upset the whole fermentative economy of the digestive tract as to displace the microbic elements proper to each district, and that this may have the effect of putting a stop to normal action of the bowels until each portion of the intestinal canal has become recharged with its normal fermentive organisms. The subject is an interesting one, and evidently has an important bearing upon the question of the cause of the aperient action of certain kinds of food which seem on analysis to contain no substance of an aperient nature. Perhaps such materials merely form a proper medium for certain micro-organisms by whose products peristalsis is kept up.

